# No replicating evidence for anti‐amyloid‐β autoantibodies in cerebral amyloid angiopathy‐related inflammation

**DOI:** 10.1002/acn3.52169

**Published:** 2024-09-13

**Authors:** Emma van den Berg, Rian Roelofs, Lieke Jäkel, Steven M. Greenberg, Andreas Charidimou, Ellis S. van Etten, Delphine Boche, Catharina J. M. Klijn, Floris H. B. M. Schreuder, H. Bea Kuiperij, Marcel M. Verbeek

**Affiliations:** ^1^ Department of Neurology, Donders Institute for Brain, Cognition and Behaviour Radboud University Medical Center Nijmegen the Netherlands; ^2^ Department of Human Genetics Radboud University Medical Center Nijmegen the Netherlands; ^3^ Department of Neurology Massachusetts General Hospital Boston Massachusetts USA; ^4^ Department of Neurology, Boston University Medical Center Boston University Chobanian & Avedisian School of Medicine Boston Massachusetts USA; ^5^ Department of Neurology Leiden University Medical Center Leiden the Netherlands; ^6^ Clinical Neurosciences, Clinical and Experimental Sciences, Faculty of Medicine University of Southampton Southampton UK

## Abstract

**Objective:**

Elevated levels of anti‐amyloid‐β (anti‐Aβ) autoantibodies in cerebrospinal fluid (CSF) have been proposed as a diagnostic biomarker for cerebral amyloid angiopathy‐related inflammation (CAA‐RI). We aimed to independently validate the immunoassay for quantifying these antibodies and evaluate its diagnostic value for CAA‐RI.

**Methods:**

We replicated the immunoassay to detect CSF anti‐Aβ autoantibodies using CSF from CAA‐RI patients and non‐CAA controls with unrelated disorders and further characterized its performance. Moreover, we conducted a literature review of CAA‐RI case reports to investigate neuropathological and CSF evidence of the nature of the inflammatory reaction in CAA‐RI.

**Results:**

The assay demonstrated a high background signal in CSF, which increased and corresponded with higher total immunoglobulin G (IgG) concentration in CSF (*r*_sp_ = 0.51, *p* = 0.02). Assay levels were not elevated in CAA‐RI patients (*n* = 6) compared to non‐CAA controls (*n* = 20; *p* = 0.64). Literature review indicated only occasional presence of B‐lymphocytes and plasma cells (i.e., antibody‐producing cells), alongside the abundant presence of activated microglial cells, T‐cells, and other monocyte lineage cells. CSF analysis did not convincingly indicate intrathecal IgG production.

**Interpretation:**

We were unable to reproduce the reported elevation of anti‐Aβ autoantibody concentration in CSF of CAA‐RI patients. Our findings instead support nonspecific detection of IgG levels in CSF by the assay. Reviewed CAA‐RI case reports suggested a widespread cerebral inflammatory reaction. In conclusion, our findings do not support anti‐Aβ autoantibodies as a diagnostic biomarker for CAA‐RI.

## Introduction

Cerebral amyloid angiopathy‐related inflammation (CAA‐RI) is an uncommon, but highly progressive and clinically relevant subtype of CAA. A pronounced perivascular inflammatory response to cerebrovascular amyloid‐β (Aβ) aggregates occurs, either with or without presence of transmural vasculitic infiltrates.^1^ This is proposed to be the result of an autoimmune reaction.^2^ CAA‐RI patients exhibit a clinically distinct appearance to sporadic CAA with a (sub)acute onset of cognitive decline, seizures, headaches, and white matter imaging abnormalities suggestive of vasogenic edema, as indicated by clinicoradiological criteria.^3^, ^4^ Brain biopsy or postmortem autopsy is required to establish a definitive diagnosis via histopathological confirmation of a local inflammatory reaction. Imaging characteristics of CAA‐RI overlap with amyloid‐related imaging abnormalities commonly observed in patients with early Alzheimer's disease after receiving anti‐Aβ immunotherapies, such as lecanemab.^4^, ^5^

Elevated levels of anti‐Aβ autoantibodies in the cerebrospinal fluid (CSF) have been proposed as a diagnostic and therapeutic biomarker for CAA‐RI.^2^ CAA‐RI patients often tend to respond very well to immunosuppressive therapy,^6^ reported to subsequently lead to a post‐treatment decrease in CSF anti‐Aβ autoantibodies.^2^ The reliability of the developed immunoassay as a specific diagnostic tool for CAA‐RI and its potential utility to quantify anti‐Aβ antibodies in monitoring immunosuppressive therapy outcomes have not been established through external validation. Additionally, radiological CAA‐RI appears to be significantly underdiagnosed,^7^ limiting timely treatment opportunities and recovery chances. Quantification of anti‐Aβ autoantibodies could enhance CAA‐RI diagnosis. Our study aimed to independently validate the immunoassay for quantifying anti‐Aβ autoantibodies and evaluate its diagnostic value as a biomarker for CAA‐RI. Moreover, we have performed an extensive literature review of published CAA‐RI case reports to unravel the exact neuropathological substrate of the inflammatory reaction in CAA‐RI.

## Methods

### CSF samples

Lumbar puncture was performed to collect CSF samples after approval by the local medical ethics committees (Arnhem‐Nijmegen, file number 2016‐3011; Leiden Den Haag Delft, file numbers P17.259 and NL63256.058.17; Southampton and South West Hampshire Local Research Ethics Committees, file number LRC 075/03/w; Partners Human Research Committee in Boston, study ID PHRC #2006P000664). We included 59 controls, 6 CAA‐RI patients, and 4 subjects who participated in the first anti‐Aβ immunization trial (AN1792, Elan Pharmaceuticals, Inc., South San Francisco, CA, USA).^8^

Fifty‐nine CSF samples (Radboud University Medical Center [RUMC], Nijmegen, the Netherlands) originated from subjects who underwent a lumbar puncture as part of a routine diagnostic assessment. Samples were randomly selected based on having a sufficient amount of CSF leftover after the completion of diagnostic tests to be used for assay characterization or comparison with CAA‐RI cases. Among those, 13 subjects did not have a neurological disorder, and for 6 subjects this information was unclear or unavailable. Of the other 40 subjects with neurological etiology of their symptoms, 27 subjects had central nervous system involvement (Table S1). Nervous system disorders were unrelated to either CAA‐RI or any other Aβ‐related neurodegenerative diseases. For a subset of control samples (*n* = 35), the total immunoglobulin G (IgG) levels in CSF were documented. Among these, 20 samples had a total IgG concentration within the reference range (<30 mg/L), whereas 15 samples had elevated total IgG levels (range: 94–457 mg/L). Total IgG levels in CSF were determined via a quantitative nephelometry standardized diagnostic test using commercially available kits and the Atellica NEPH 630 System (Siemens Healthcare GmbH, Marburg, Germany).

Three subjects from Leiden (Leiden University Medical Center, the Netherlands), one subject from Boston (Massachusetts General Hospital, Boston, MA, USA), and one subject from Nijmegen (RUMC) fulfilled diagnostic criteria of probable CAA‐RI.^3^ The sixth subject originated from Boston and was assigned a diagnosis of definitive CAA‐RI via brain biopsy. Five of these six CAA‐RI CSF samples were collected during the acute disease phase, whereas the sixth sample (with definitive CAA‐RI) was collected 1 month after initiation of immunosuppressive therapy. For the other 2 cases that received immunosuppressive therapy, CSF was collected prior to the treatment initiation. The time between symptom onset and CSF sampling varied for the included CAA‐RI cases. This ranged from 3 days after symptom onset, to 3 months after (see Table S1 for all details on clinical background).

CSF samples from the four AN1792 participants were collected postmortem (Southampton General Hospital, Southampton, United Kingdom). Three of them developed variable anti‐Aβ titers in serum unrelated to CAA‐RI, while the fourth received a placebo treatment. These samples were included as a separate informative group. Although information regarding anti‐Aβ titers in CSF was unavailable, it could be expected that the serum levels were mirrored in CSF, considering the brain is the primary site of action for the applied immunotherapy.

Demographic and clinical details on all included samples, including which corresponding figures their data is presented, are provided in Table S1.

### Anti‐Aβ autoantibodies assay

The assay to detect anti‐Aβ autoantibodies in human CSF has previously been described.^2^, ^9^ We have exactly followed this procedure to replicate the original findings. In brief, total IgG is obtained by immunoprecipitation (IP) using protein G‐coupled magnetic beads (Dynabeads Protein G; Thermo Fisher Scientific, Waltham, MA, USA, cat: 10004D) from 500 μL CSF, of which 110 μL eluate was collected (i.e., 4.5x concentrated). Then, the quantified immunoassay concentration (in ng/mL) was immediately measured by an enzyme‐linked immunosorbent assay (ELISA). Microwell plates were coated with 0.5 μg of Aβ_1‐42_ peptide (AnaSpec, Inc., San Jose, CA, USA, cat: AS‐20276), and monoclonal mouse anti‐Aβ antibody (clone 4G8; Biolegend, San Diego, CA, USA, cat: 800701) was used as standard in the calibration curve. All samples were assayed in duplicate (50 μl eluate per well). Supernatants after IP (i.e., fraction of CSF samples that did not bind to protein G beads) were also assayed to check for IP efficiency. Standards and samples were incubated overnight at 4°C. The next day, the plate was incubated with horseradish peroxidase conjugate protein A (Merck‐Millipore, Burlington, MA, USA, cat: 18–160) for 2 h, followed by incubation with 3,3′,5,5′‐tetramethylbenzidine for 20 min in the dark, both at room temperature. The reaction was stopped by adding 1 N H_2_SO_4_, and the absorbance was read at 450 nm using a microplate reader. In between steps, the plate was washed three times with PBS/0.05% Tween‐20. One protocol modification was made regarding the quantity of magnetic beads used in the IP to minimize experimental costs: we have reduced this quantity from 75 μL to 50 μL. Figure S1 shows that the optimal amount of beads was 50‐75 μL, which both yielded comparable results in the ELISA. To confirm that the assay can detect human anti‐Aβ antibodies we also used the recombinant humanized monoclonal anti‐Aβ antibody aducanumab (Thermo Fisher Scientific, Waltham, MA, USA, cat: MA5‐42013). Moreover, to test specificity of the results, we replaced the Aβ_1‐42_ coating with nonsense scrambled Aβ_1‐42_ peptide coating (AnaSpec, Inc., San Jose, CA, USA, cat: AS‐25382) or reversed Aβ_40‐1_ peptide coating (AnaSpec, Inc., San Jose, CA, USA, cat: AS‐22817). We assessed the protein concentration of the peptide stocks using a Bradford assay (Sigma‐Aldrich, St. Louis, MO, USA, cat: B6916).

### Data processing and statistical analyses

Data was analyzed using GraphPad Prism software version 9.5.0 (GraphPad Software, Inc., San Diego, CA, USA). Calibration curves were created by generating a four parameter logistic curve fit. Shapiro–Wilk test was used to assess data normality. Statistical analyses were performed using a Student's *t*‐test or analysis of variance with Tukey's *post hoc* test, as appropriate. Pearson's or Spearman's correlations were used to assess associations between variables, as appropriate. Significance threshold was set at *p* < 0.05. Variables are presented as means with standard deviations.

### Literature review of reported CAA‐RI cases

Available literature was reviewed to assess the neuropathological substrate of CAA‐RI and investigate cellular evidence for the production of anti‐Aβ autoantibodies. A PubMed search was conducted using “*cerebral amyloid angiopathy”* and *“inflammation”* as the keywords. Publications up to November 2023 were screened. We applied no restrictions during the literature search, since many different nomenclatures are generally used to describe CAA‐RI (e.g., inflammatory CAA, CAA with related inflammation, and perivasculitis), especially before the publication of diagnostic criteria.^3^, ^4^ Titles and abstracts were reviewed for case reports on CAA‐RI (or any of the formerly listed alternatives) that assessed relevant inflammatory markers in CSF or neuropathology. Data were collected for cases for which individual information was available.

## Results

### Assay performance

Calibration curves generated using 4G8 and aducanumab yielded comparable results, although a slightly steeper curve for aducanumab was observed (Fig. S2A). A signal comparable to the blank was observed when either antibody was applied to scrambled Aβ_1‐42_ coating. When aducanumab was spiked in human CSF (concentrations: 6.25–100 ng/mL before IP), after the IP procedure, a dose‐dependent increase in signal was observed when using Aβ_1‐42_ coating, as expected. However, a high background signal was observed using non‐spiked CSF and a substantial nonspecific signal was consistently observed using spiked CSF and scrambled Aβ_1‐42_ coating, similar to non‐spiked CSF (Fig. S2B).

We noticed that in the assay a considerable signal above the blank signal was obtained for CSF samples (*n* = 14) on scrambled Aβ_1‐42_ coating compared to normal Aβ_1‐42_ coating (Fig. S3A). The mean signal on scrambled Aβ_1‐42_ coating was consistently 51% (range: 38–60%) of the signal obtained when using Aβ_1‐42_ coating, demonstrating a strong correlation (*r*_p_ = 0.98, *p* < 0.0001). Similar outcomes were observed when using reversed Aβ_40‐1_ instead of scrambled Aβ_1‐42_ peptide as a coating (*n* = 5 CSF samples; Fig. S3B). The mean signal for CSF on reversed Aβ_40‐1_ peptide coating was 68% (range: 62–73%) of that on normal Aβ_1‐42_ peptide coating, with a strong correlation between results on both coatings (*r*_p_ = 0.99, *p* = 0.0008).

### Quantified immunoassay concentrations in CSF from CAA‐RI and controls

**Figure 1:**
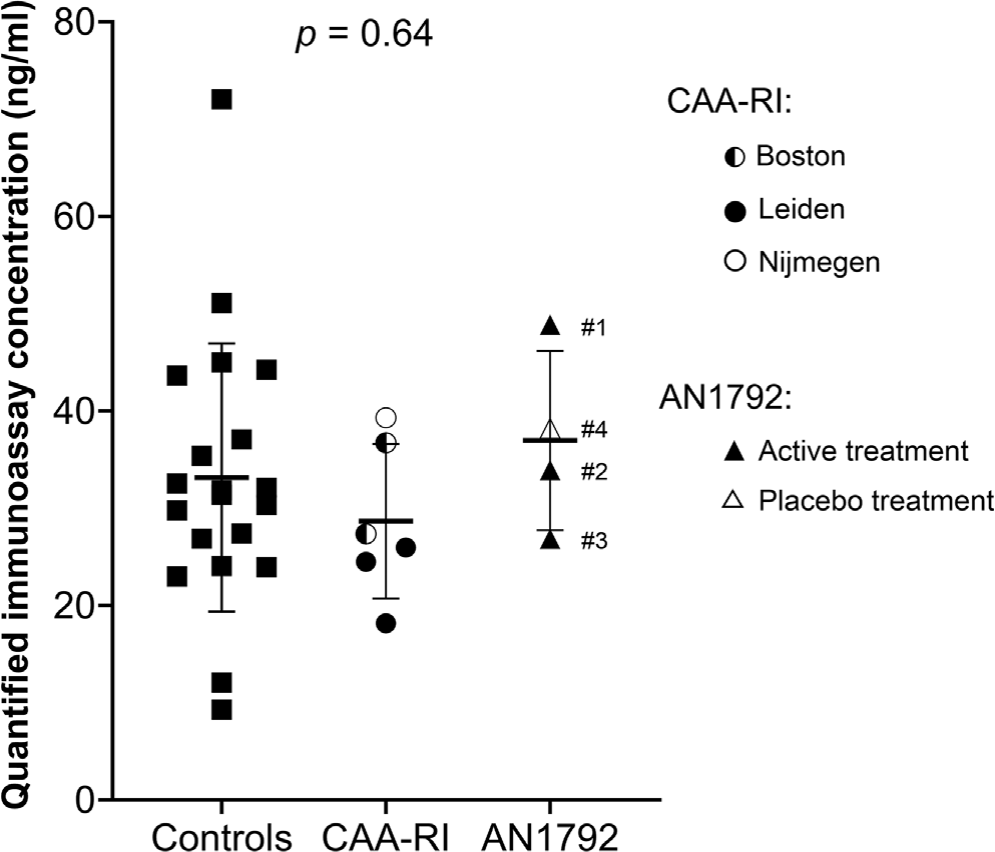
Quantified immunoassay concentration in control subjects and patients. Displayed are the quantified immunoassay concentrations (ng/mL) in the cerebrospinal fluid (CSF) of control subjects (*n* = 20), cerebral amyloid angiopathy‐related inflammation (CAA‐RI) patients (*n* = 6), and subjects that participated in the AN1792 active amyloid‐β (Aβ) immunization trial (*n* = 4).^8^ CSF from one subject who received placebo treatment (open triangle) was not included in the analysis of variance (ANOVA). Concentrations were similar for all three groups (*p* = 0.64; ANOVA). Mean values and standard deviations are indicated. The legend indicates center of origin for CAA‐RI samples and the treatment type for AN1792 trial participants. Sample numbers for the latter are specified in the figure. The anti‐Aβ plasma titers for these samples were: 1707 for sample #1, 142 for samples #2 and #3, 0 for sample #4.

The mean immunoassay concentration quantified by the assay was 33.2 ng/mL (±13.8) in controls (*n* = 20), 28.7 ng/mL (±7.9) in CAA‐RI patients (*n* = 6), and 37.0 ng/mL (±9.2) in AN1792 participants (*n* = 3; participant receiving placebo was not included). The participant receiving placebo treatment in the AN1792 trial displayed an intermediate quantified immunoassay concentration of 38.2 ng/mL. The levels were comparable for all three groups (*p* = 0.64; Fig. 1).

### Correlation of quantified immunoassay concentration with total IgG levels in CSF

**Figure 2:**
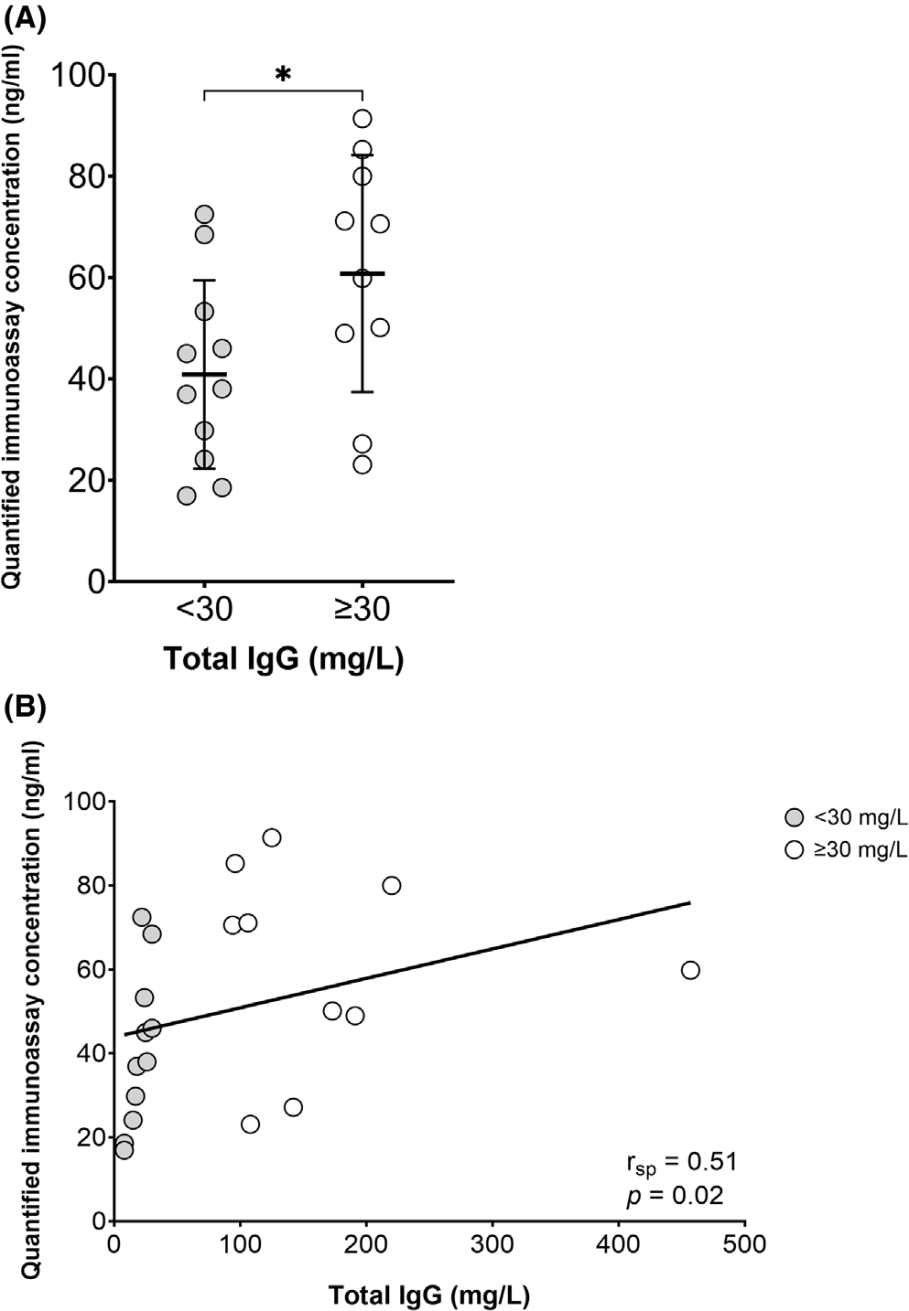
Relationship between quantified immunoassay concentrations and total immunoglobulin G levels in cerebrospinal fluid of control subjects. (A) Quantified immunoassay concentrations (ng/mL) in the cerebrospinal fluid (CSF) of control subjects with normal (<30 mg/L; *n* = 11) or elevated (≥30 mg/L; n = 10) total immunoglobulin G (IgG) CSF levels. Quantified immunoassay concentrations were increased in subjects with elevated total IgG levels (*p* = 0.04; t‐test). Asterisk indicates a significant *p* value. Mean values and standard deviations are indicated. (B) Spearman's correlation (*r*_sp_) between quantified immunoassay concentrations and total IgG levels in the CSF of control subjects. A positive correlation was observed (*r*_sp_ = 0.51, *p* = 0.02).

Since we did not observe elevated quantified immunoassay concentrations in CSF from CAA‐RI patients, we hypothesized that previously reported elevations were due to increased total IgG levels in CSF and nonspecific detection of IgG in the assay. Therefore, we investigated a potential relationship between the quantified immunoassay concentration and the total IgG level in CSF samples. When applying a cut‐off value of <30 mg/L (i.e., the upper limit of reference value for CSF IgG in our lab), individuals with a normal total IgG level (*n* = 11) displayed lower quantified immunoassay concentrations, compared to those with elevated (*n* = 10) total IgG CSF levels (*p* = 0.04; Fig. 2A). A significant correlation was observed between the quantified immunoassay concentration and total IgG levels (*r*_sp_ = 0.51, *p* = 0.02; Fig. 2B). Moreover, we have found that the quantified immunoassay concentrations using the standard protocol lead to an underestimation of the true concentration in samples with elevated IgG content. Of these samples specifically, the IP supernatants (i.e., unbound IgG fraction) still displayed levels of IgGs not bound to the protein G beads, resulting in a positive signal in the assay. These IgGs were not extracted by the protein G beads from the input CSF sample. In fact, the proportion of residual IgGs in supernatants was notably higher in samples with elevated total IgG levels compared to those with normal total IgG levels (Fig. S4).

### Literature review of reported CAA‐RI cases

Eighty‐two case reports comprising descriptions of a total of 135 patients with CAA‐RI, including individual case data on neuropathology or CSF, were included in the current study. Extracted information from all cases and all included case reports, are described in Table S2, and a summary of the results is provided in Table 1. The mean age of the cases was 69.9 ± 9.4 years and 66 (49.3%) were female. The large majority of patients (*n* = 124, 91.9%) were treated with anti‐inflammatory therapy. Of those, 119 patients received corticosteroid therapy (93.7%), and additional immunosuppressive therapy was administered to 10 (7.9%) patients. The initial outcome after treatment was clinical and/or neuroimaging improvement (*n* = 75 of 93 reported, 80.6%), lack of improvement (*n* = 6, 6.5%), or further clinical deterioration (*n* = 12, 12.9%), and not reported in the remaining patients (*n* = 31).

**Table 1:** Summary of neuropathological and cerebrospinal fluid findings from 135 case reports on CAA‐RI from 82 publications.

	*N* of *N* reported	Percentage of *N* reported
Sex	134^a^	
Female	66/134	49.3%
Male	68/134	50.7%
Anti‐inflammatory treatment	127^a^	
Corticosteroids	119/127	93.7%
Other immunosuppressive	10/127	7.9%
Treatment outcome	93^a^	
Improvement	75/93	80.6%
Stable	6/93	6.5%
Deterioration	12/93	12.9%
Neuropathology		
Tissue examination	69^a^	
Brain biopsy	60/69	87.0%
Autopsy	12/69	17.4%
Lymphocytes	59^a^	
T‐cells	43/59	72.9%
B‐cells	2/28	7.1%
None	16/59	27.1%
Inflammation location	64^a^	
Perivascular	51/62	82.3%
Transmural vasculitis	16/55	29.1%
Vessel wall destruction	42^a^	
Present	9/42	21.4%
Absent	33/42	78.6%
Granulomatous infiltration	45^a^	
Macrophages	21/45	46.7%
Histiocytes	9/29	31.0%
None	20/44	45.5%
Giant cells	49^a^	
Mononucleated	17/49	34.7%
Multinucleated	20/49	40.8%
None	23/49	46.9%
Gliosis	54^a^	
Microglia	25/54	46.3%
Astrocytes	12/54	22.2%
None	26/54	48.1%
CSF analysis	95^a^	
Pleocytosis (WBC >4)	33/88	37.5%
Elevated total protein (≥450 mg/L)	72/90	80.0%
Unique oligoclonal IgG bands	4/26	15.4%

CAA‐RI, cerebral amyloid angiopathy‐related inflammation; CSF, cerebrospinal fluid; IgG, immunoglobulin G; *N*, number of; WBC, white blood cell.

^a^ Numbers of patients that were analyzed for each characteristic.

Neuropathological analysis was available for 69 patients (51.5%). Sixty (87.0%) underwent a brain biopsy, and for 12 (17.4%) patients their tissue was analyzed at autopsy (3 subjects underwent both). Exact sampling location was only rarely reported (*n* = 20, 29.0%), but if so, the location of tissue sampling was always at an abnormal lesion site typical for CAA‐RI based on neuroimaging. Lymphocytic involvement consisting of perivascular T‐cells was frequently observed (*n* = 43 of 59 reported, 72.9%), and only very rarely of B‐cells (*n* = 2 of 28 reported, 7.1%). Plasma cells were never reported. Perivascular inflammatory characteristics were present in 51 patients (62 reported, 82.3%), whereas 16 (55 reported, 29.1%) displayed transmural vasculitis. Disruption of vessel walls was present in 9 (21.4%) of the 42 reported patients. Granulomatous infiltration was present in 25 patients (45 reported, 53.3%) and consisted of either macrophages (*n* = 21 of 45 reported, 46.7%) or histiocytes (*n* = 9 of 29 reported, 31.0%). The majority of patients displayed mononucleated (*n* = 17 of 49 reported, 34.7%) or multinucleated (*n* = 20 of 49 reported, 40.8%) giant cells. Gliosis was observed in 28 patients (54 reported, 51.9%), with microglia present in 25 (of 54 reported, 46.3%) and astrocytes in 12 (of 54 reported, 22.2%).

Almost two‐fifth of the patients for whom white blood cell count in CSF was reported had pleocytosis (cut‐off >4; *n* = 33 of 88 reported, 37.5%). Elevated protein levels (cut‐off ≥450 mg/L) were present in 72 patients (90 reported, 80.0%). Unique oligoclonal IgG bands in the CSF were found in a fraction (*n* = 4 of 26 reported, 15.4%) of patients.

## Discussion

The main findings of our study are as follows. (1) In the CSF of CAA‐RI patients the levels of anti‐Aβ antibodies, as quantified by a previously described assay, were not found to be elevated compared to non‐CAA‐RI controls. (2) Signal obtained in this assay was attributable to nonspecific binding of IgG. (3) Extensive neuropathological analysis of CAA‐RI case reports revealed abundant presence of glial cells, T‐lymphocytes, and other monocyte lineage cells. Collectively, our data do not replicate previous publications stating that CSF levels of anti‐Aβ autoantibodies can be detected and serve as a suitable diagnostic biomarker for CAA‐RI.

Earlier reports employing this assay described elevated concentrations of anti‐Aβ autoantibodies in the CSF of CAA‐RI patients.^2^, ^5^, ^10^, ^11^, ^12^, ^13^, ^14^ In contrast, we observed comparable quantified immunoassay concentrations in both CAA‐RI patients and controls, and could thus not replicate this finding. Notably, the majority of earlier reported autoantibodies concentrations (11 of 13 described CAA‐RI cases^5^, ^10^, ^11^, ^12^, ^13^) fall within the quantified immunoassay concentration range that we observed in our non‐CAA control CSF samples. Moreover, most of the previous reports^5^, ^10^, ^12^, ^13^, ^14^ did not include a direct comparison with control subjects, introducing uncertainty regarding whether CAA‐RI levels deviated at all from controls. Additionally, the quantified immunoassay concentrations did not reflect the anticipated rank order of AN1792 participants, deviating from the expected pattern based on their documented anti‐Aβ plasma titers (e.g., subject with a plasma titer of 0 did not have the lowest quantified immunoassay concentration).

We demonstrated that the anti‐Aβ assay faithfully detects specific mouse (4G8 clone) and humanized (aducanumab) anti‐Aβ antibodies. Importantly, however, when applied to CSF samples enriched for total IgG by IP, the assay yielded a high level of nonspecific signal, as demonstrated by the high background levels when using wild‐type or nonsense Aβ peptides as coating, and by the fact that quantified immunoassay levels correlated with total IgG levels in CSF. Despite the capability of the assay to detect and quantify mouse and humanized Aβ antibodies, both the IP efficiency and ELISA proved to be insufficiently specific to demonstrate the anti‐Aβ antibodies. Analysis of CSF samples depleted for IgG by IP indicated that not all IgGs were successfully extracted from the samples, particularly for samples with elevated total IgG levels. This is not unexpected since the binding capacity of protein G magnetic beads is exceeded (capacity equals maximally 12.5–15 μg of human IgG per 50 μL beads) for samples with total IgG concentrations >30 mg/L. This warrants the use of a higher volume of magnetic beads in the IP. Nevertheless, an impasse has been reached as using larger volumes of magnetic beads hampered the IgG yield of the IP for samples with normal total IgG levels (see Fig. S1), and thus arguing against the fact that larger bead volumes may increase the IP efficacy. Moreover, the applied IP method does not enrich the sample for anti‐Aβ antibodies but instead concentrates all IgG by a factor of 4.5. Analysis of the immunodetection part of the assay highlights several crucial shortcomings thereby creating a nonspecific assay. Applied CSF samples yielded a consistently high background signal when using Aβ_1‐42_ or the nonsense peptide coatings scrambled Aβ_1‐42_ or reversed Aβ_40‐1_. Nonspecific binding of antibodies to nonsense peptides has been observed more often, especially in serum samples with high IgG concentrations.^15^ Low‐affinity binding of immunoglobulin subclasses to arbitrary peptides or polystyrene surfaces may explain our observations. Given the correlation of the quantified immunoassay concentrations with total IgG CSF levels, and with such large amounts of nonspecific IgG applied to the immunodetection, it is not surprising that a fraction of nonspecific IgG will bind to Aβ_1‐42_. Notably, the discrepancy in signal observed between Aβ_1‐42_ and scrambled Aβ_1‐42_ is likely caused by distinct fractions of random IgG, each displaying different affinities for nonspecific binding to the respective (nonsense) Aβ peptides. In summary, it is likely that nonspecific IgG binding contributed to the assay's high background readings.

The extensive analysis of neuropathological case reports indicated that perivascular leukocytes were primarily of the T‐lymphocyte type, and antibody‐producing B‐lymphocytes^16^ were only very scarcely observed. The observation that a variety of immune cells are widely observed in CAA‐RI tissue samples (such as macrophages, histiocytes, mononucleated or multinucleated giant cells, astrocytes, and microglia), and the generally positive effects of immunosuppressive therapy, supports the existence of a cerebral inflammatory reaction in CAA‐RI, though not necessarily an antibody‐mediated inflammatory response. The review of case reports also indicated that elevated total protein concentrations in CSF were prominent in the large majority of CAA‐RI patients. The integrity of the blood–brain barrier is known to be compromised in CAA,^17^ and the CSF/blood albumin quotient correlates with the number of cerebral microbleeds in CAA (our own unpublished data) and Alzheimer's disease patients.^18^ The permeability of the blood–brain barrier is even more pronounced in CAA‐RI, leading to fluid extraversion on brain magnetic resonance imaging (i.e., vasogenic edema) and extraversion of erythrocytes (i.e., microbleeds).^1^ This indicates that systemic IgGs may leak from the blood into the CSF^19^ via the blood–brain barrier or blood‐CSF barrier and contribute to the elevated IgG levels in CSF.^11^

Limitations of our study include the inevitable absence of neuropathological confirmation in five out of six CAA‐RI patients. However, the current diagnostic criteria for possible and probable CAA‐RI have proven their accuracy in differentiating CAA‐RI patients from noninflammatory sporadic CAA,^4^ as well as the oftentimes considered differential diagnosis of primary angiitis of the central nervous system,^20^ without the necessity of histopathological confirmation. Moreover, control samples were not prospectively included, leading to a diverse range of disease etiologies and varying origins of symptoms. However, our analysis in CAA‐RI samples versus these controls demonstrated the non‐specificity of the assay. One CAA‐RI sample was obtained approximately 1 month after initiation of immunosuppressive therapy, which could have influenced the quantified immunoassay concentration, although the conclusion of the statistical analysis after exclusion of this sample remained unchanged (data not shown). Our literature study is subjected to publication and observer biases. Also, it is essential to acknowledge that lack of certain neuropathological characteristics in case reports does not necessarily indicate their absence in a patient. Underreporting should be considered when interpreting the review findings. The lack of a neuropathological description could either imply its absence in a patient, or that it has not been examined at all.

In conclusion, the overall performance of the anti‐Aβ autoantibody assay was unsatisfactory in terms of specificity and efficiency. We did not find compelling evidence that the assay detects anti‐Aβ autoantibodies or that such levels are increased in CAA‐RI. The lack of replicating evidence for the presence of anti‐Aβ autoantibodies do not support their use as a biomarker for the diagnosis of CAA‐RI.

## Author Contributions

EB, LJ, HBK, and MMV designed the study. SMG, AC, ESE, DB, CJMK, and FHBMS were responsible for recruitment of patients and collection of (clinical) patient data. RR performed the experiments. EB, RR, LJ, HBK, and MMV interpreted the data. EB performed the literature review and wrote the manuscript draft. All authors critically revised and contributed to the manuscript draft, and read and approved the final manuscript.

## Conflict of Interest

The authors declare that they have no conflicts of interest to report.

## Supporting information


Figure S1.

Table S1.

## Data Availability

The datasets used and/or analyzed in the current study are available from the corresponding author upon reasonable request.
