# Supplementary material for: No replicating evidence for anti‐amyloid‐β autoantibodies in cerebral amyloid angiopathy‐related inflammation
Source: Ann Clin Transl Neurol. 2024 Sep 13;11(10):2563–71. doi: 10.1002/acn3.52169 (PMC11514902; doi:10.1002/acn3.52169)
Supplement: Supplementary file 1 — Figure S1. [file ACN3-11-2563-s001.docx]

**Supplementary files**

**No replicating evidence for anti-amyloid-β autoantibodies in cerebral amyloid angiopathy-related inflammation**

Emma van den Berg, MSc^1^, Rian Roelofs^2^, Lieke Jäkel, PhD^1^, Steven M. Greenberg, MD, PhD^3^, Andreas Charidimou, MD, PhD^4^, Ellis S. van Etten, MD, PhD^5^, Delphine Boche, PhD^6^, Catharina J.M. Klijn, MD, PhD^1^, Floris H.B.M. Schreuder, MD, PhD^1^, H. Bea Kuiperij, PhD^1^, Marcel M. Verbeek, PhD^1,2,*^

Affiliations:

1. Department of Neurology, Donders Institute for Brain, Cognition and Behaviour, Radboud University Medical Center, Nijmegen, the Netherlands
2. Department of Human Genetics, Radboud University Medical Center, Nijmegen, the Netherlands
3. Department of Neurology, Massachusetts General Hospital, Boston, Massachusetts, USA
4. Department of Neurology, Boston University Medical Center, Boston University Chobanian & Avedisian School of Medicine, Boston, Massachusetts, USA
5. Department of Neurology, Leiden University Medical Center, Leiden, the Netherlands
6. Clinical Neurosciences, Clinical and Experimental Sciences, Faculty of Medicine, University of Southampton, Southampton, United Kingdom

*Corresponding author. Department of Neurology, Radboud University Medical Center, 830 TML, P.O. Box 9101, 6500 HB, Nijmegen, The Netherlands. E-mail: [marcel.verbeek@radboudumc.nl](mailto:marcel.verbeek@radboudumc.nl)

**Supplementary tables**

Both supplementary tables are available in the provided Excel file.

**Supplementary Table S1.** Characteristics of all included cerebrospinal fluid samples.

**Supplementary Table S2.** Extracted information from all case report studies included in the literature review.

**Supplementary Figures**


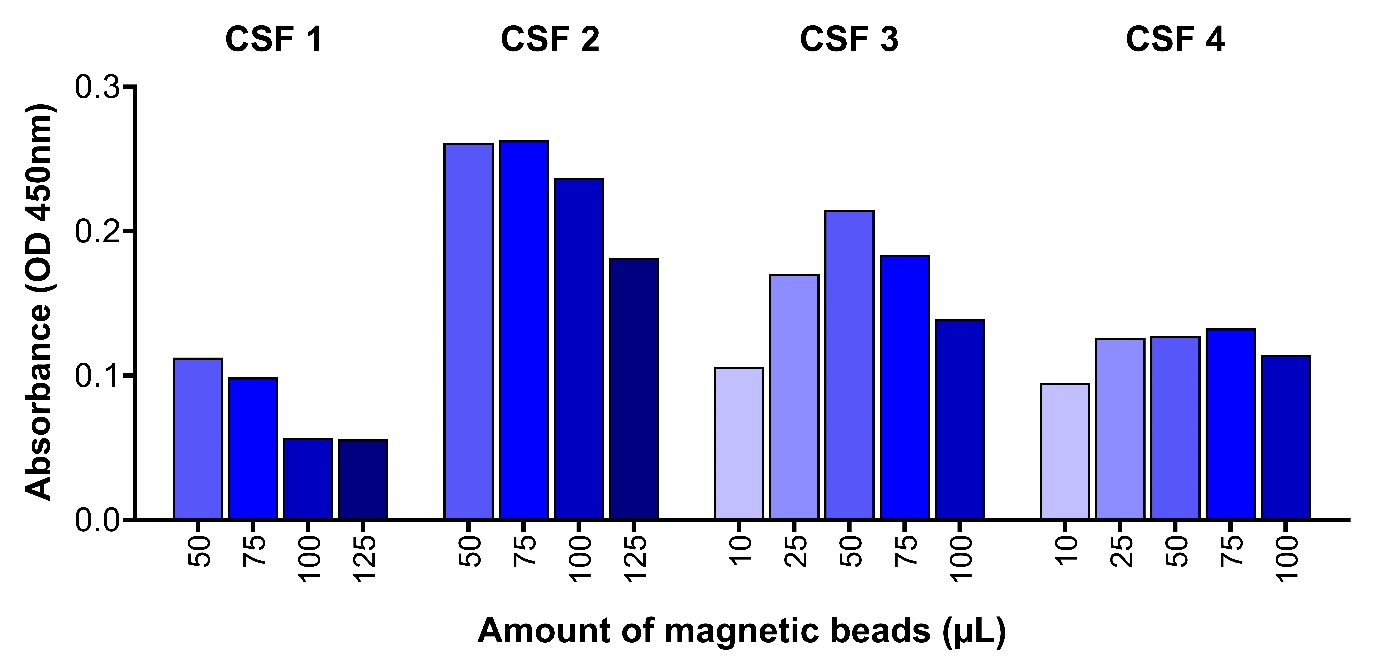


**Supplementary Figure S1. Optimization of magnetic beads quantity used in immunoprecipitation.** The use of different amounts of magnetic beads (10-25-50-75-100-125µL) were tested in the immunoprecipitation (IP), applied to four different random cerebrospinal fluid (CSF) samples, and subsequently followed by one-sided enzyme-linked immunosorbent assay (ELISA) analysis. Both 50µL and 75µL beads in the IP yielded very similar optical density (OD) values in the ELISA for all CSF samples. A smaller amount of beads decreased the obtained signal, whereas increasing the amount above 75µl also resulted in a lower signal, potentially attributable to steric hindrance. Fifty microliters of beads were chosen as optimal and cost-effective amount for the IP reaction.

**
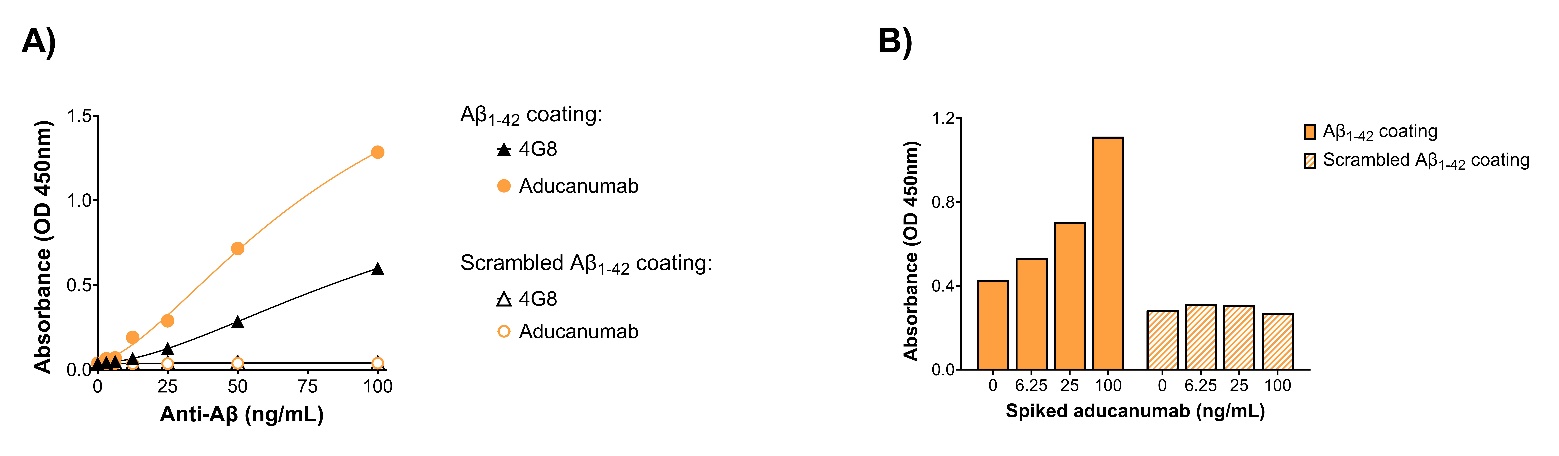
Supplementary Figure S2. Aducanumab and 4G8 performance in ELISA.** **(A)** Calibration curves for 4G8 (black) and aducanumab (orange) antibodies on amyloid-β_1-42_ (Aβ_1-42_) coating (closed shapes) or scrambled Aβ_1-42_ coating (open shapes). For both antibodies, on Aβ_1-42_ coating a valid calibration curve was obtained. A signal comparable to blank was present for both antibodies on scrambled Aβ_1‑42_ coating (overlapping curves in figure). **(B)** Results of CSF spiked with different concentrations of aducanumab before immunoprecipitation (0-100 ng/mL), followed by analysis in enzyme-linked immunosorbent assay (ELISA) on Aβ_1-42_ or scrambled Aβ_1-42_ coating. The quantified signal of cerebrospinal fluid (CSF) samples on scrambled Aβ_1-42_ coating could only be expressed in terms of optical density (OD) values, rather than concentration, since the calibration curve displayed no signal on the scrambled Aβ_1-42_ coating. A dose-dependent increase in signal was observed on Aβ_1-42_ coating (solid orange). Consistent high background signal was observed on scrambled Aβ_1-42_ coating, independent of the spiked aducanumab concentration (dashed orange).


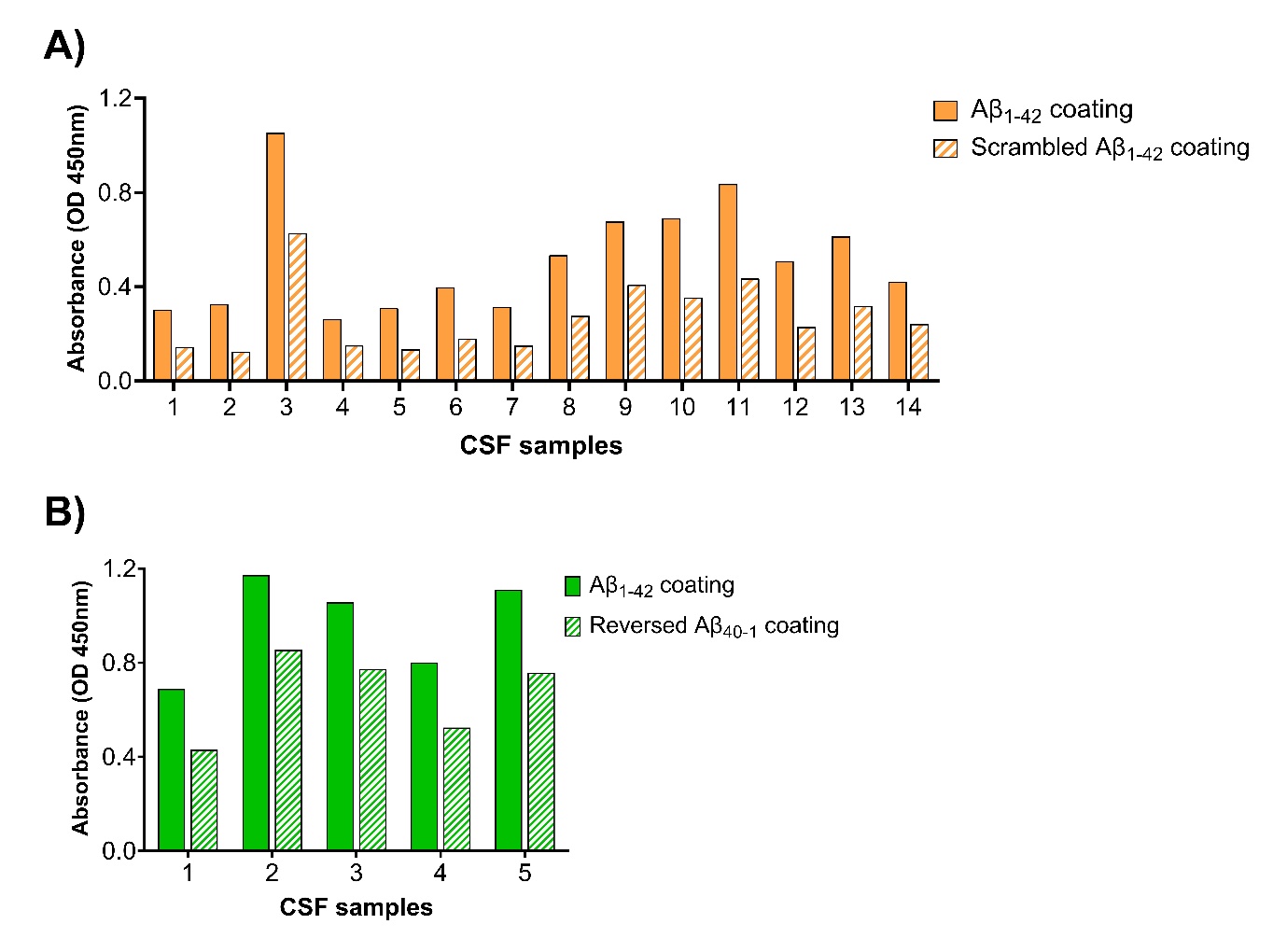


**Supplementary Figure S3. Cerebrospinal fluid sample analysis using different peptide coatings in the ELISA.** Displayed are the obtained signals for random cerebrospinal fluid (CSF) samples on amyloid-β_1-42_ (Aβ_1-42_) coating (orange and green solid bars) in comparison with scrambled Aβ_1-42_ coating (orange dashed bars) or reversed Aβ_40-1_ coating (green dashed bars). Equal peptide concentrations were coated, based on protein quantification by Bradford assay. A consistent high signal was obtained using **(A)** the scrambled Aβ_1-42_ peptide coating (51% of that obtained with Aβ_1‑42_ coating), or **(B)** the reversed Aβ_40-1_ peptide coating (68% of that obtained with Aβ_1‑42_ coating), for all CSF samples. On both nonsense coatings the samples exhibited a comparable signal pattern to the Aβ_1‑42_ coating.


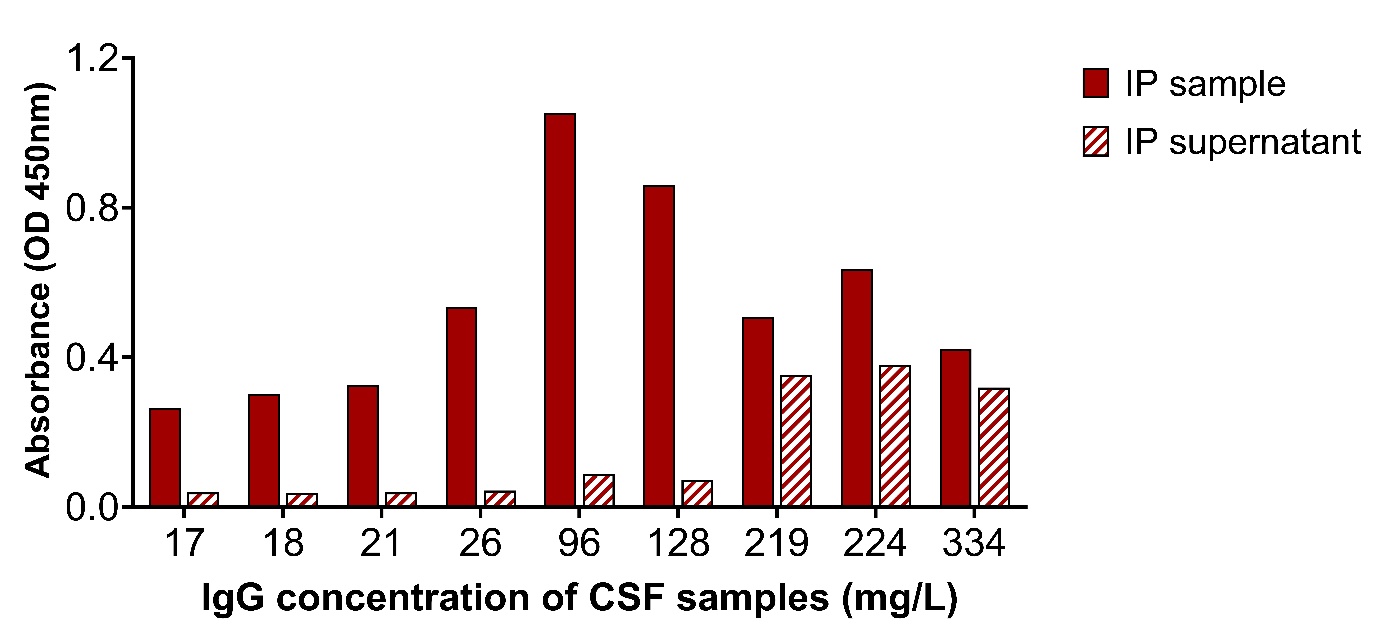


**Supplementary Figure S4. Efficiency of immunoprecipitation procedure.** Displayed are the obtained signals for random cerebrospinal fluid (CSF) samples with normal (<30 mg/L) or elevated total immunoglobulin G (IgG) concentrations (≥30 mg/L) on amyloid-β_1-42_ coating after the immunoprecipitation (IP) procedure. Solid bars represent the enriched immunoprecipitated sample. Dashed bars represent the supernatant, remaining after the IP, containing the unbound fraction. It can be appreciated that there is more residual target not precipitated from CSF samples containing very high (*i.e.*, more than 200 mg/L) total IgG levels compared to the IP of samples with low total IgG levels, indicating a less efficient IP result in the former samples.
